# Recent Advances on Drug-Loaded Mesenchymal Stem Cells With Anti-neoplastic Agents for Targeted Treatment of Cancer

**DOI:** 10.3389/fbioe.2020.00748

**Published:** 2020-07-23

**Authors:** Amirhesam Babajani, Pegah Soltani, Elham Jamshidi, Mohammad Hadi Farjoo, Hassan Niknejad

**Affiliations:** ^1^Department of Pharmacology, School of Medicine, Shahid Beheshti University of Medical Sciences, Tehran, Iran; ^2^Student Research Committee, School of Medicine, Shahid Beheshti University of Medical Sciences, Tehran, Iran; ^3^Student Research Committee, School of Pharmacy, Shahid Beheshti University of Medical Sciences, Tehran, Iran

**Keywords:** mesenchymal stem cell, cancer, chemotherapeutic drugs, targeted therapy, angiogenesis, metastasis, apoptosis, proliferation

## Abstract

Mesenchymal stem cells (MSCs), as an undifferentiated group of adult multipotent cells, have remarkable antitumor features that bring them up as a novel choice to treat cancers. MSCs are capable of altering the behavior of cells in the tumor microenvironment, inducing an anti-inflammatory effect in tumor cells, inhibiting tumor angiogenesis, and preventing metastasis. Besides, MSCs can induce apoptosis and inhibit the proliferation of tumor cells. The ability of MSCs to be loaded with chemotherapeutic drugs and release them in the site of primary and metastatic neoplasms makes them a preferable choice as targeted drug delivery procedure. Targeted drug delivery minimizes unexpected side effects of chemotherapeutic drugs and improves clinical outcomes. This review focuses on recent advances on innate antineoplastic features of MSCs and the effect of chemotherapeutic drugs on viability, proliferation, and the regenerative capacity of various kinds of MSCs. It also discusses the efficacy and mechanisms of drug loading and releasing procedures along with *in vivo* and *in vitro* preclinical outcomes of antineoplastic effects of primed MSCs for clinical prospection.

## Introduction

Cancer is an inflammatory disease that is known as abnormal cell growth with the ability to invade and metastasize to a distance from a primary tumor site. Approximately 1,700,000 new cases of cancer are diagnosed in the United States each year, which is equivalent to more than 4,800 cases per day (Siegel et al., 2019). Establishment of new treatment protocols in recent years can result in a lower mortality rate of cancer patients. There are a variety of methods to treat cancer; all of them aim to whether suppress tumor growth or inhibit metastasis. Chemotherapy is one of the best known therapeutic choices. The effect of chemotherapeutic drugs on tumor tissue depends on several factors, especially the route of injection and physiological barriers around the tumor tissue. These drugs can be injected systemically or locally and affect both tumor and normal cells, hence the name collateral toxicity. Therefore, there are many efforts in developing targeted delivery methods with less toxic effects on normal tissues of the body. One of the promising approaches for targeted cancer therapy is using stem cells as both a therapeutic agent and a drug delivery vehicle. Among different types of stem cells, mesenchymal stem cells (MSCs) have characteristic features that facilitate their use in targeted therapy of cancer. MSCs naturally have intrinsic antitumor activities, which include antiproliferative effects, suppressing angiogenesis, decreasing metabolisms, and inducing apoptosis. MSCs are able to modulate immune reactions against themselves and evade the immune system, which makes them capable of being circulated in blood vessels without inducing immune response. They are also able to differentiate into a variety of adult cell types, which makes them capable of reconstructing the damaged tissues after interventions such as surgery (Koç et al., 2000; Uccelli et al., 2006; Rhee et al., 2015; Lee and Hong, 2017).

Mesenchymal stem cells can originate from different human sources. Based on the International Society for Cellular Therapy classification, MSCs include human bone marrow–derived MSCs (hBM-MSCs), human adipose-derived MSCs (hAD-MSCs), human dental-derived MSCs (hD-MSCs), human olfactory bulb neural stem cells (Hu-OBNSCs), and human placenta and umbilical cord–derived MSCs. Approximately 1% of human white adipose tissue consists of hAD-MSCs. There are two main sources of white adipose tissue in human: first, subcutaneous fat in the abdomen, gluteus, and thighs; and second, abdominal fat around gastrointestinal tract, omentum, and perineum (Ong and Sugii, 2013). MSCs can also be isolated from different parts of teeth and gingiva. Human dental-derived MSCs originate from dental pulp, exfoliated deciduous teeth, periodontal ligament, apical papilla, dental follicle, and gingiva (GinPa-MSCs) (Huang et al., 2009; Coccè et al., 2017). MSCs could be isolated from different parts of the placenta and umbilical cord including amniotic membrane (AM-MSCs), chorionic plate, decidua parietalis, and umbilical cord (Wu et al., 2018). MSCs express some common cell markers such as CD73, CD90, and, CD105 but they do not express CD34, CD45, CD14, CD11b, CD79-α, CD19, and HLA-DR (Vidal et al., 2012; Ong and Sugii, 2013; El-Bialy et al., 2014; Gay et al., 2014; El-Sayed et al., 2015; Jin et al., 2015; Yin et al., 2016; Kuci et al., 2019). Although MSCs from different sources display the mentioned common markers, they possess some exclusive characteristics. For example, some types of MSCs possess the capacity to form colonies and differentiate into multilineage cells such as neurons, endothelial cells (Zhang Q. et al., 1950), and myocardial-like cells (Huang et al., 2015). The International Society for Cellular Therapy has considered osteoblastic, adipocytic, and chondrocytic differentiation capacity as minimal criteria to characterize MSCs. As a phenotypic criterion, plastic-adherent capacity in standard culture conditions helps to distinguish MSCs (Dominici et al., 2006).

The important characteristic that makes MSCs superior to the other cells is low immunogenicity. MSCs do not express high levels of major histocompatibility complex (MHC) class II and CD40 (Uccelli et al., 2006; Li et al., 2015). It has been shown that MSCs are immune evasive. This feature makes them a proper candidate for transplantation and migration inside the body when injected intravenously and makes them able to track tumors efficiently without being affected by immune system. Moreover, these cells are able to decrease both initiation and/or progression of tumors through the modulation of immune responses. Several types of cancer tend to occur in the sites of chronic inflammation and tissue damage (Multhoff et al., 2011). This correlation is defined as two main categories: intrinsic and extrinsic pathways. In the intrinsic pathway, genetic factors stimulate activation of protooncogenes and inactivation of tumor suppressor genes, which results in normal cell transformation into abnormal cells and subsequent inflammation. In the extrinsic pathway, the risk of cancer development increases subsequent to a chronic inflammation or infection in the high-risk organs such as prostate and skin. In both pathways, an increase in the production of proinflammatory molecules, cytokines, and interleukins (ILs) stimulates activation and recruitment of different immune cell types (Multhoff et al., 2011). MSCs suppress the early inflammation after exposure to a carcinogen agent. MSCs decrease the infiltration of macrophages to the site of inflammation up to 50% and reprogram these cells to involve in phagocytosis rather than producing proinflammatory cytokines (Francois et al., 2019). MSCs also decrease the expression of both proinflammatory mRNAs and proteins [IL-1α, IL−1β, IL−4, IL-5, IL-6, IL-12, MIP-2, tumor necrosis factor α (TNF−α), and interferon γ (IFN-γ)] and increase the amount of anti-inflammatory mRNAs and proteins [IL-10 and transforming growth factor β (TGF-β)] in the site of inflammation (Tang et al., 2015; Francois et al., 2019). MSCs restore the C-reactive protein concentration in the blood to its basal levels, which is a marker of systemic immune response against carcinogenic agents (Francois et al., 2019). These cells stimulate expression of regulatory T cell (Treg) phenotype, which selectively suppress effector T cells and play an important role in limiting the cell-mediated immune response. MSCs secrete TGF-β, which activates Smad-2. The phosphorylation of the latter factor results in higher amounts of foxp3, which is the transcription factor of Treg cells (Tang et al., 2015). As a result, expression of TGF-β mRNA by MSCs is coherent with higher expression of Treg cells, and their accumulation in lymph nodes suppresses excessive and chronic inflammation before tumor formation and improves patient prognosis (Tang et al., 2015). In addition to inhibition of tumor initiation in sites of chronic inflammation and reducing tumor size, MSCs can limit fibrosis after radiation therapy of tumors and increase survival of the animal models of cancer after irradiation of the tumor site (Francois et al., 2019). MSCs also seem to possess special features, which make them an appropriate choice to be used as drug carrier. They can be loaded with several anticancer molecules such as chemotherapeutic drugs, which can be released in the tumor microenvironment after tumor homing. This type of drug delivery increases the anticancer drug efficacy on tumor cells and decreases collateral toxicity.

In this review, we will summarize recent studies on the inherent anticancer property of MSCs, their resistance against antitumor drugs and involved mechanisms of this resistance, MSCs’ capacity of uptaking/releasing the antineoplastic drugs and their related mechanisms, metabolism of anticancer drugs in MSCs, and effectiveness of drug-loaded MSCs in cancer therapy.

## Routes of Delivery and Tumor Homing of MSCs

It seems that multiple injections of MSCs directly into the tumor can provide a high number of cells in tumor microenvironment and cause acceptable result (Seo et al., 2011). Single-dose intratumoral injection of MSCs into the pancreatic ductal subcutaneous adenocarcinoma in athymic mice causes approximately 50% reduction in size and weight of the tumor. MSCs were observed around peripheral vasculature and necrotic areas of the tumor (Cousin et al., 2009). Intratumoral administration of MSCs in the subcutaneous induced melanoma model resulted in apoptosis of endothelial cells (Otsu et al., 2009). However, this method of administration requires several invasive interventions, which increases the risk of infection and cannot treat far metastases of the primary tumor site. Besides, repeated intratumoral administration cannot be practical in deep tumors and short-period therapeutic courses. In order to surmount problems of direct injection, application of catheter-based delivery of therapeutic cells can be a choice to deliver MSCs into the deep tumors (Parker Kerrigan et al., 2017). Furthermore, application of drug carriers such as exosomes and nanoparticles is an alternative option to deliver drugs to tumor site. Exosomes are able to cross biological membranes including the blood-brain barrier (BBB), and they show very low unspecific interaction with circulating blood proteins (El Andaloussi et al., 2013; Liao et al., 2019). However, tumor homing inability and providing appropriate sources and amounts of exosomes for clinical application are still main problems (Nawaz et al., 2016; Luan et al., 2017). Nanoparticles are able to carry high amounts of multiple drugs and protect their content from external damage (Jiang and Gao, 2017). Nevertheless, low targeting capacity, toxic effects, and fast clearance from circulation are some bottlenecks of their application (Liao et al., 2019). Considering these concerns, it is critical to find more efficient routes of cell or drug delivery to achieve proper therapeutic goals. The ability of MSCs to migrate to the sites of inflammation and tumor microenvironment makes them suitable to be used by delivery routes other than intratumoral injection.

Systemically injected MSCs can efficiently home to tumor sites. Secretion of different proinflammatory molecules in the tumor sites, including IFN-γ, TNF-α, IL-6, IL-8, TGF-β, hepatocyte growth factor, platelet-derived growth factor (PDGF), vascular endothelial growth factor (VEGF), and CXCL12 and some other chemoattractant molecules (Seo et al., 2011), prompts circulating MSCs to migrate to tumor sites (D’Souza et al., 2013). Precise mechanism of MSC infiltration to the tumor site is not fully understood, but studies have suggested that it is a combination of three main mechanisms. Similar to wounded tissues, several cytokines and chemokines are released in the tumor microenvironment, whose receptors are expressed on the MSC membrane. Monocyte-chemoattractant protein 1 (CCL2), VEGFα, and PDGFαβ are well known to be highly expressed in tumor microenvironment and attract MSCs to the site of tumor (Ball et al., 2007; Dwyer et al., 2007). Expression of chemokine receptors on MSC membrane (e.g., chemokine receptor 4) is influenced by the features of tumor microenvironment (hypoxia and TNF-α), which makes such cytokine/receptor pair reactions more specific for MSC migration to the tumor site (Ponte et al., 2007; Kidd et al., 2009; Otsu et al., 2009). In the second mechanism, it is suggested that metabolic status of tumor microenvironment attracts MSCs. For example, hypoxia increases the expression of MCP-1 through production of nitric oxide and hypoxia-induced transcription factor 1α (Spaeth et al., 2008). Another mechanism involved in MSC migration to tumor site is expression of adhesion molecules on the cell membrane of MSCs. Several adhesion molecules including vascular cell adhesion molecule 1 (VCAM-1), intercellular adhesion molecule 1/3 (ICAM-1/3), activated leukocyte cell adhesion molecule, endoglin, and several subtypes of Toll-like receptors are expressed on the cell membrane of MSCs. These molecules are also expressed by leukocytes, dendritic cells, and monocytes, which suggest a similar mechanism of migration to the site of inflammation for MSCs (Spaeth et al., 2008). These studies have suggested that the highest amount of MSCs migration happened in the presence of the three mechanisms combined.

Based on tumor tracking ability, one of the most appropriate methods for delivery is systemic injection of MSCs in which vascular system delivers cells to tumor and metastasis sites. Different routes of injection have been used to evaluate the efficacy of each of them. It has been reported that intravenous (i.v.) injection resulted in the accumulation of human MSCs (hMSCs) in metastatic melanoma (Tyciakova et al., 2017), glioma (Kosztowski et al., 2009), and colorectal tumor sites (Kucerova et al., 2007). One hour after injection of hMSCs into the femoral vein and the common carotid artery of the rat with established orthotropic glioblastoma, hMSCs appeared in the peripheral zone of glioblastoma where angiogenesis is prominent. By injection of hMSCs into the femoral vein, 0.02% of injected hMSCs accumulated at the glioblastoma site. Common carotid artery and the internal carotid artery injections were 0.1 and 0.5%, respectively. The ipsilateral injection of hMSCs to brain glioblastoma was significantly more efficient than intracardiac injection. It seems, the closer injection sites to a tumor, the better therapeutic outcome.

One of the challenges that should be bypassed to deliver MSCs to brain is BBB. MSCs have the ability to cross this barrier in certain conditions (Akiyama et al., 2002; Chen et al., 2003; Osaka et al., 2010). MSCs express chemokine receptors (e.g., CXCR4, CCR2) and cell adhesion molecules (e.g., CD44, integrins α4 and β1, and CD99) on their surface, which are important during MSC adhesion to BBB endothelial cells in sites of damage and inflammation (Ji et al., 2004). Several *in vitro* studies have been done to clarify the role of other molecules such as TIMP3 (Menge et al., 2012), VCAM-1, and *P*-selectin during the initial rolling steps of MSC homing (Teo et al., 2012).

Intraperitoneal infusion is another route that can be used to deliver hMSCs to tumor sites. In a metastatic mouse model of ovarian carcinoma, intraperitoneal administration of hMSCs showed localization of these cells in three to four spots by 7 days, and the number of hMSCs remained unchanged for 1 more week (Kidd et al., 2009). Compared with i.v. administration, intraperitoneal injection resulted in better localization of hMSCs in neuroblastoma site, whereas a majority of i.v.-injected hMSCs remained in the lungs for a while (Kimura et al., 2016). Although lung as a filter organ can trap systemically injected hMSCs and postpone cell access to target tumors, hMSCs are able to leave the lungs gradually and appear in tumor sites. There are no mortality or major side effects (e.g., pulmonary embolism) for hMSC injection (Pacioni et al., 2017).

Intranasal delivery is a less invasive method that can increase patient comfort and compliance. Application of hMSCs through nasal cavity shows that these cells can rapidly penetrate nasal cavity wall and enter brain tissues. It seems that quick penetration is related to direct path of hMSCs through the trigeminal and olfactory pathways. In addition, it is reported that irradiation can increase hMSC concentration in tumor sites (Balyasnikova et al., 2014).

Meningeal metastasis is a common problem among neoplasms of central nervous system. Intrathecal administration of MSCs is an appropriate option to eliminate leptomeningeal metastatic glioma. Engineered MSCs can reduce the size of established leptomeningeal glioma up to 80% and prolong the life span of intrathecally injected mice. After injection of MSCs into the cerebellomedullaris cistern, these cells migrated to the peritumoral area and deep parts of established leptomeningeal glioma (Gu et al., 2010).

**FIGURE 1:**
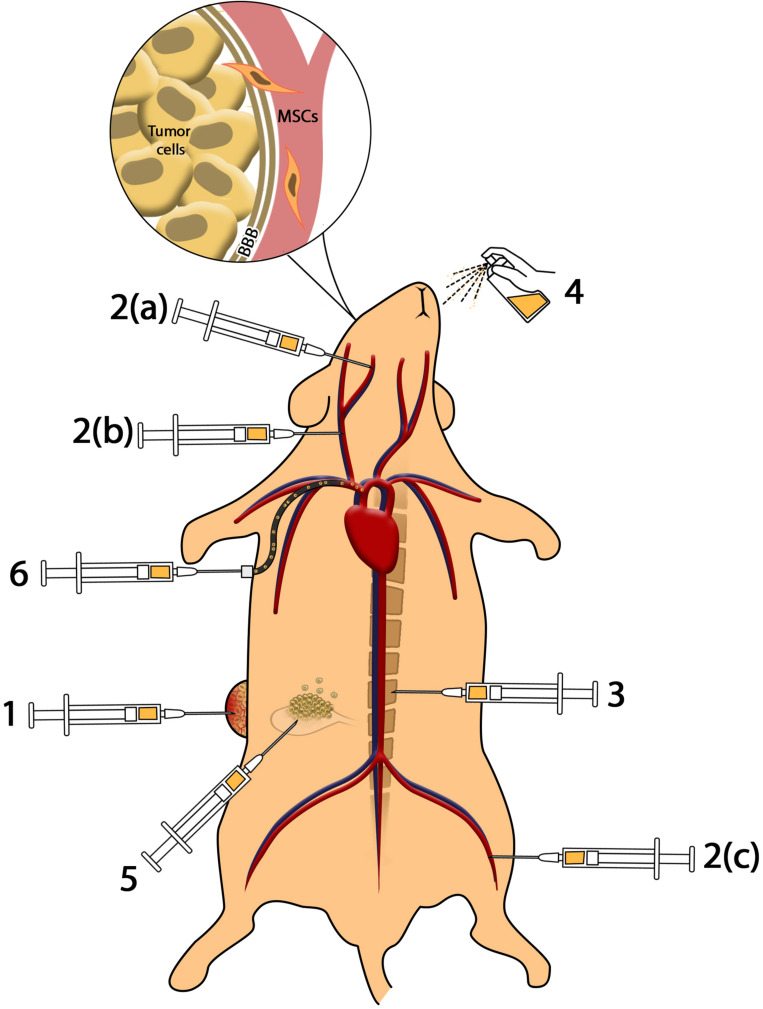
Methods of cell delivery. **(1)** Intratumoral injection provides higher amounts of MSCs in the tumor microenvironment; however, impressive complications including infection, pain, and accessibility to deep tumors reduce the efficacy. **(2)** Intravenous and intra-arterial injection: (a) injection of MSCs to internal carotid artery results in accumulation of MSCs in brain tumors such as glioblastoma; (b) injection of MSCs in common carotid artery reduces the efficacy of cell delivery to glioma in comparison to internal carotid artery; (c) MSCs injected through the femoral vein enter cardiopulmonary circulation that reduces the efficacy of cell administration. Intravenous and intra-arterial injected MSCs cross the BBB to reach brain malignancies. **(3)** Intrathecal administration enables MSCs to access cerebrospinal fluid (CSF) and reach meningeal tumors. **(4)** Intranasal administration of MSCs as a novel method reduces complications of injection and provides MSCs in brain tumors. **(5)** Intraperitoneal injection of MSCs causes distribution in peritoneal cavity and can be used in ovarian malignancies. **(6)** Application of catheter-based cell delivery provides a safe pathway to deliver MSCs to deep organs and reduce the complication of direct injection.

Different routes of cell/drug delivery and their efficacy are shown in Figure 1.

## Effects of MSCs on Tumors

In the tumor microenvironment, MSCs interfere with intracellular mechanisms of tumor cells, which control their metabolism and growth. They may reduce tumor cell proliferation (Kuci et al., 2019), angiogenesis, and migration to other tissues and metastases and/or increase their apoptosis. MSCs exert these effects by down-regulating essential signaling pathways for tumor progression such as Wnt, Notch, Shh, and BMP pathways (Imitola et al., 2004; Ponte et al., 2007; Karp and Leng Teo, 2009; Momin et al., 2010; Dai et al., 2013; Naderi-Meshkin et al., 2016; Francois et al., 2019). For example, coculture of MSCs with hepatoma cells results in an increase in tumor cell apoptosis and decrease in proliferation by down-regulating Bcl-2, c-Myc, proliferating cell nuclear antigen (PCNA), and survivin protein levels in hepatoma tumor cells (Lowe et al., 2010); all of them are targets of Wnt signaling (Gordon and Martinez, 2010; Ji et al., 2016). MSCs can inhibit Akt protein kinase in Kaposi sarcoma cells, which is an essential enzyme in multiple cellular processes such as glucose metabolism, apoptosis, cell proliferation, transcription, and cell migration.

Akt promotes Forkhead box O (FoxO) 3a, which regulates transcription of several genes that participate in tumor apoptosis cell cycle progression, DNA repair, oxidative stress resistance, and other cellular functions (Chen J. et al., 2010; Chen Q. et al., 2010; Yang et al., 2010; Lam et al., 2012; Ruvolo, 2012; Shukla et al., 2013, 2014; Wang et al., 2013; Zhang Q. et al., 2020). Understanding the most important mechanisms by which MSCs affect tumors helps us properly manipulate these cells for future translation into the clinic.

### Inducing Apoptosis

Mesenchymal stem cells can increase tumor cell apoptosis by suppressing Akt phosphorylation. They increase PTEN (a negative regulator of Akt activation) in tumor cells, which results in higher accumulation of FoxO3 in tumor cells. FoxO3 stimulates the extrinsic pathway of apoptosis by up-regulating death receptor expression including Fas ligand and TNF-related apoptosis-inducing ligand (TRAIL) (Ramasamy et al., 2007). MSCs express Fas ligand on their surface and stimulate the extrinsic pathway of apoptosis in tumor cells through Fas/Fas ligand connection. This connection results in the up-regulation of caspase-3 and caspase-8 enzymes (Di Germanio et al., 2016). Human adipose-derived MSCs when cocultured with T-cell lymphoma model cells *in vitro* down-regulated inactive procaspase-3 and up-regulated poly (ADP-ribose) polymerase (PARP) in tumor cells. PARP (a group of proteins involved in DNA repair) depletes cell ATP while trying to fix DNA damages, and this depletion results in cell death (Ahn et al., 2014). MSCs also induce impairment in mitochondrial function, which is known by an increase in the Bax/Bcl-2 and Bax/Bcl-xL ratio and loss of mitochondrial membrane potential (MMP). These events coinciding with caspase activation stimulate the intrinsic pathway of apoptosis (Willert and Jones, 2006).

### Inhibition of Proliferation (Cell Cycle Arrest)

Treatment of tumor cell lines with MSCs has resulted in a decrease in Ki67 expression in tumor cells, which is a marker of cell proliferation (Francois et al., 2019). MSCs affect the expression of several regulators of cell transition between the phases of cell cycle and as a result inhibit cell transition between different phases, which results in lower proliferation levels. MSCs are able to decrease expression of positive regulators of cell cycle including regulators of G1 phase and G1/S transition (CCNE, CCNH, CCND2, CDK2, CDK4, CDK6, CUL1, SKP2, RBL1), S phase and DNA replication (MCM2, MCM3, MCM4, MCM5, PCNA, DDX11), G2 phase and G2/M transition (CCNH, CDK5R1, DDX11)(Magatti et al., 2012; Bu et al., 2016).

Mesenchymal stem cells up-regulate cell cycle inhibitory genes including inhibitors of G1 phase and G1/S transition (CCNG2, CDKN1A, CDKN2B, RB1), G2 phase and G2/M transition [CDKN1A; CCNE1: cyclin E1; CCNH: cyclin H; CCND2: cyclin D2; CDK: cyclin-dependent kinase; CUL1: Cullin 1; SKP2: S-phase kinase-associated protein 2 (p45); MCM: minichromosome maintenance complex component; PCNA: proliferating cell nuclear antigen; DDX11: DEAD/H (Asp-Glu-Ala- Asp/His box polypeptide 11); CDK5R1: cyclin-dependent kinase 5, regulatory subunit 1 (p35); RBL1: Retinoblastoma-like 1 (p107); CCNG1: cyclin G1; CCNG2: cyclin G2; CDKN1A: cyclin-dependent kinase inhibitor 1A (p21, Cip1); CDKN2B: cyclin-dependent kinase inhibitor 2B (p15, inhibits CDK4); RB1: Retinoblastoma 1] (Magatti et al., 2012; Bu et al., 2016). For example, FoxO3a inhibits cancer cell progression from G1 to S phase by up-regulating cell cycle inhibitory proteins p21 and p27 (Bu et al., 2016), whereas angiostatin and thrombospondin, which are highly expressed in the hAM-MSCs, can increase the number of cancer cells in G1 phase and decrease the number of cells in G2/M phase and S phase and, as a result, inhibit their further proliferation (Ramasamy et al., 2007; Rolfo et al., 2014; Di Germanio et al., 2016; Modaresifar et al., 2017).

Although the lower number of cells is enough for suppressing tumor cell proliferation when MSCs and tumor cells are in direct contact (Bu et al., 2016), a part of cell cycle arrest is related to the secreted molecules from MSCs. The antitumor effects of hAM-MSCs were evident even when MSCs and cancer cells were physically separated using a Transwell membrane (Bu et al., 2016). It is noteworthy that blocking these paracrine signaling pathways, using RNA interference or neutralizing antibodies against antitumor secretions of MSCs, does not suppress the antiproliferative effects of MSC on tumor cells (Zhu et al., 2009), which suggests that the antiproliferative effect of MSCs is through complex paracrine/direct contact-dependent mechanisms.

### Inhibition of Angiogenesis

Although MSCs are mostly known for their angiogenesis potential through a variety of secreted molecules, they can efficiently suppress angiogenesis in tumors both *in vivo* and *in vitro* and, as a result, increase focal necrosis in solid tumors (Adelipour et al., 2017). This antiangiogenesis effect may be a result of direct contact between MSC and endothelial cell or may be a result of MSC interaction with cancer cells. Human bone marrow–derived MSCs are able to migrate to capillary walls and intercalate between endothelial cells in capillary network of tumor and connect to endothelial cells through connexin 43. These cells transfer their mitochondria to endothelial cells as a subsequence of the fusion of two cells in order to shape gap junctions through connexin molecules (Otsu et al., 2009). These mitochondria are activated in the target cell and increase the production of reactive oxygen species (Hendrata and Sudiono, 2019) and induce apoptosis in endothelial cells (Otsu et al., 2009). Therefore, it seems that the antiangiogenic effect of MSCs on endothelial cells is dependent on the direct contact between these two types of cells and MSCs/endothelial cells ratio; the higher the number of MSCs, the higher endothelial cells death (Otsu et al., 2009). MSCs also increase the expression of caspase-3 enzyme or activate FasL-dependent pathway in endothelial cells and promote their apoptosis and in turn suppress angiogenesis (Hendrata and Sudiono, 2019).

Cell–cell contact of MSCs with endothelial cells of tumor induces cell cycle arrest in endothelial cells, as mentioned in cancer cells. They decrease the number of cells in S phase, and this effect was dependent on the concentration of MSCs in the culture environment. They also increase the number of cells in G1 phase with no effect on G2/M phase. The cell cycle arrest by MSCs occurs when there is only direct contact between endothelial cells and MSCs (Menge et al., 2013).

Mesenchymal stem cells also resulted in lower expression of IL-1β and cathepsin B in tumor cells. The latter factor is highly expressed in tumor cells, and its down-regulation results in suppression of endothelial progenitor cell mobilization and recruitment to make new vessels in tumor site (Malla et al., 2010). MSCs also reduce the expression of several molecules in tumor such as PDGF, which play an important role in inducing angiogenesis. Platelet-derived growth factor–BB/PDGF receptor β interaction, which is one of the pathways of endothelial progenitor cell mobilization (Gerhardt and Betsholtz, 2003; Bergers and Song, 2005), was suppressed in glioma endothelial cells when cocultured with MSCs, as a result angiogenesis and tumor size reduced in the glioma tumor model (Ho et al., 2013).

### Inhibition of Metastasis

Mesenchymal stem cells have the capability to inhibit tumor metastasis. As mentioned before, Akt is an important pathway in tumor progression and metastasis. MSCs up-regulate PTEN, which decreases the amount of phosphorylated Akt. Phosphorylation of Akt is related to tumor metastasis by activating MMP enzymes, which are necessary during extracellular matrix degradation within tumor metastasis (Dasari et al., 2010). MSCs also decrease tumor cell motility (Dasari et al., 2010). This effect may be through increasing expression of intercellular adhesion molecules, for example, *E*-cadherin and vimentin, which play an important role in tumor cell stabilization in its primary site. MSCs also inhibit epithelial-to-mesenchymal transition (EMT), which is an essential mechanism during tumor metastasis. During EMT, tumor cells obtain new mesenchymal-like features including increased motility.

Presence of MSCs when cocultured with glioma cells reduces the number of pericytes in the tumor microenvironment (Ho et al., 2013). Pericytes are groups of cells that play important role in the maintenance of vessel integrity, and their absence is associated with a higher level of permeability of vessels for tumor cells and a subsequent higher level of metastasis (Xian et al., 2006; Gerhardt and Semb, 2008).

**FIGURE 2:**
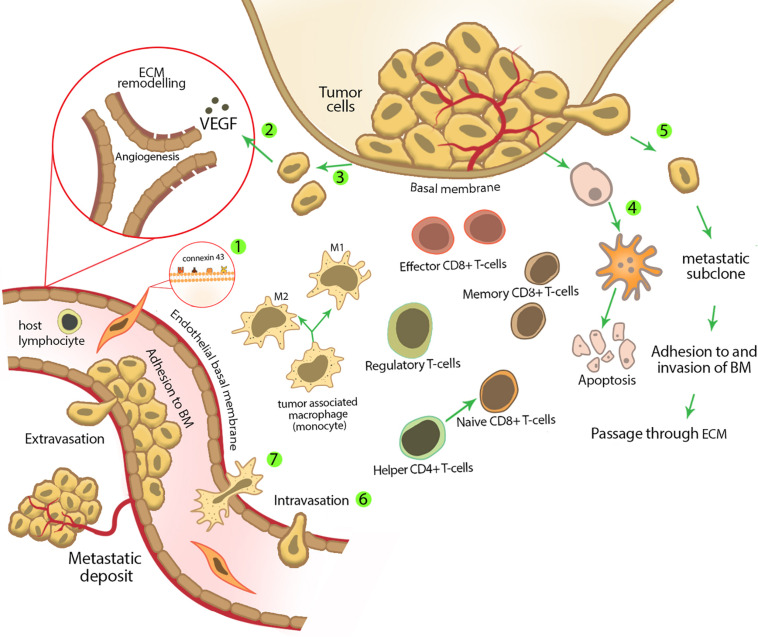
Mesenchymal stem cell characteristics and activities in tumor suppression. **(1)** Mesenchymal stem cells express several surface molecules that have a pivotal role in their homing to tumor sites. These surface molecules are important during MSC adhesion to different cell types; for example, MSCs express connexin 43 on their membrane, which plays an important role in MSC adhesion to endothelial cells. **(2)** Tumor cells are able to produce several growth factors, for example, vascular endothelial growth factor, which stimulates new vessel formation and angiogenesis. Mesenchymal stem cells inhibit tumor angiogenesis by reducing the secretion of these growth factors and inducing apoptosis and/or cell cycle arrest in endothelial cells. **(3)** During and after tumor formation, tumor cells undergo uncontrolled proliferation. Mesenchymal stem cells inhibit tumor cell proliferation by decreasing the expression of positive regulators of cell cycle and/or regulating cell cycle inhibitory genes. **(4)** Mesenchymal stem cells can increase tumor cell apoptosis either by up-regulating death receptors expression (extrinsic pathway of apoptosis) or stimulating intrinsic pathway of apoptosis. **(5)** Mesenchymal stem cells inhibit tumor metastasis by decreasing tumor cell motility in the primary site of a tumor. **(6)** Decreasing permeability of lymphatic or blood vessels for circulating tumor cells. **(7)** Early after carcinogen exposure, the initial inflammatory response is started, which recruits other innate immune cells from nearby capillaries. Mesenchymal stem cells affect different types of immune cells in the site of inflammation and modulate the immune response, which results in tumor inhibition. Mesenchymal stem cells decrease the amount of M2 phenotype macrophages (which promote tumor tolerance and angiogenesis through secretion of VEGF, TGF-β, and other soluble factors) and induce more regulatory T cells (which are produced during the active phase of immune response and limit the strong immune response by CD4^+^ and CD8^+^ cells and as a result prevent damage to the host tissue) to enter the inflammatory site.

Mesenchymal stem cells are able to inhibit secondary tumor cells in sites of metastasis. For example, in Ewing sarcoma model (the most common bone tumor among children), i.v. injection of MSCs inhibited tumor growth in the metastatic site through homing into primary and secondary tumor sites (Hayes-Jordan et al., 2014). We summarized the anticancer effect of MSCs in Figure 2.

## Protumorigenic Effects of MSCs

It has been shown that MSCs sometimes promote tumor progression. Protumorigenic effects of MSCs can be explained in two main categories, direct cell–cell contact and paracrine effects. MSCs can be recruited by tumor and secrete a variety of cytokines and growth factors in the tumor microenvironment, which promote tumor progression through facilitating tumor angiogenesis, modulating antitumor immune responses, and increasing tumor resistance against antitumor drugs. In the other words, the metabolic features of the tumor microenvironment and inflammatory cytokines (which are released from tumor microenvironment resident cells) change MSCs’ features and secretory profile, and as a result, they are no more similar to primary MSCs outside the tumor site. For example, tumor microenvironment has a lower tension of oxygen in comparison with normal tissues, called hypoxia. Chronic hypoxia both improves the protective role of MSCs for endothelial progenitors and changes the secretome of MSCs (Liu et al., 2015). The altered MSCs induce progression in the metastasis, angiogenesis, and macrophage recruitment by tumor cells and also inhibit immune cells infiltration to the tumor site (Rivera-Cruz et al., 2017). It has been shown that expression of indolamine 2,3-dioxygenase was increased in MSCs cocultured with breast cancer cells, which resulted in suppression of immune response by decreasing infiltration of CD3^+^ and CD8^+^ T lymphocytes and CD57^+^ natural killer cells to the tumor site, and also increases the number of regulatory CD4^+^ T cells (Ino et al., 2008; Bahrami et al., 2017). MSCs have been also shown to improve tumor progression, after injection to the tumor site, through secretion of several growth factors such as TGF-β, epidermal growth factor receptor, periostin, ANG1, PDGF, insulinlike growth factor, and IL-6 (Shangguan et al., 2012; Ye et al., 2012; Akimoto et al., 2013; Lee et al., 2013; Lin et al., 2013; Kansy et al., 2014; Hossain et al., 2015; Wolfe et al., 2016; Wang et al., 2018; Wang Y. et al., 2019). Some studies have suggested that direct contact of MSCs with tumor cells is necessary for exerting their tumor progression roles. For example, MSCs cocultured with breast cancer cells overexpressed CCL5, which stimulates tumor cell motivation and metastasis, whereas this effect was not evident in MSCs separated from tumor cells with a permeable membrane (Karnoub et al., 2007). In addition, MSCs cocultured with cancer cells changed into tumor-associated fibroblasts, which have a pivotal role in tumor stromatogenesis and progression of metastasis (Spaeth et al., 2009).

In order to reduce protumorigenic effects of MSCs and provide more effective therapeutic approaches, some interventional methods have been suggested such as cell engineering, genetic manipulation, and drug loading (Dwyer et al., 2010; Huang et al., 2013). Drug-loaded MSCs inhibit angiogenic factors such as Willebrand factor, CD31 (PECAM1), VEGF-α, Ve-cad, TGF-β1, CD44, and αSMA. Moreover, drug-loaded MSCs are able to inhibit ICAM1, VCAM1, and VEGF, which have a pivotal role in EMT and metastasis (Pessina et al., 2013).

## MSCs in Drug Delivery Systems

Studies suggested that the application of MSCs as a drug delivery system resulted in a better antineoplastic effect. Gemcitabine-loaded MSCs suppress the proliferation of ductal pancreatic adenocarcinoma more effectively than non-loaded MSCs. In the equal ratio of MSCs and tumor cells, the inhibitory effect of non-loaded MSCs approximately was 15%, whereas that of drug-loaded MSCs was approximately 90% (Bonomi et al., 2017b). An *in vivo* study showed that MSCs loaded with paclitaxel induced strong inhibition of lung metastasis of murine melanoma. Considering the drug content of each cell, this antimetastatic effect was equal to 2,000-fold higher amounts of pure paclitaxel (Nicolay et al., 2016b). Application of MSCs as a drug carrier system provides several advantages in comparison to usual drug administration methods. These advantages include targeted drug delivery to tumor and metastatic cells, reduced side effects of chemotherapeutic drugs, increase in drug half-life, and decrease in administered drug amount. To use MSCs as a drug delivery system, these cells must possess special features besides their innate anticancer capability. Thus, it is essential to evaluate the effects of chemotherapeutic drugs on biological aspects of MSCs such as viability, proliferation, differentiation, and reconstructive ability.

### Effects of Chemotherapeutic Drugs on MSCs

#### Effect of Chemotherapeutic Drugs on Viability of MSCs

As the first influential factor, it is important to evaluate the effect of loaded chemotherapeutic drugs on the viability of MSCs. The type of drug and MSC are two critical factors that affect the sensitivity of MSCs to antineoplastic drugs. Gemcitabine (Bonomi et al., 2015b) and bortezomib (Bonomi et al., 2017b) induce very low cytotoxicity in hBM-MSCs even in concentrations higher than 10,000 ng/mL. These cells showed moderate reduction in viability, in 3,000 ng/mL of cisplatin (Nicolay et al., 2016a) and 10,000 ng/mL of bleomycin (Nicolay et al., 2016b). Human bone marrow–derived MSCs demonstrated low death rate in 10,000 ng/mL of paclitaxel (Pessina et al., 2011, 2013; Bosco et al., 2015; Pascucci et al., 2014; Bonomi et al., 2017b), whereas in concentrations less than half of that (4,000 ng/mL), they showed a moderate cytotoxicity (Münz et al., 2018). Some drugs may be more cytocidal for hBM-MSCs at lower concentrations. Sorafenib causes 40% cell death at 465 ng/mL (Clavreul et al., 2017). Cytarabine, daunorubicin, and vincristine even in very low concentrations significantly induce apoptosis in hBM-MSCs (Nicolay et al., 2016b; Somaiah et al., 2018).

Human AD-MSCs are moderately resistant to cisplatin (Gilazieva et al., 2016; Rimoldi et al., 2018), cationic platinum (II)-complex (Rimoldi et al., 2018), vincristine, and camptothecin (Liang et al., 2011). The viability of hAD-MSCs after exposure to cisplatin and camptothecin for 3 days was more than 70%. Paclitaxel up to 10,000 ng/mL resulted in only 20% cell death in hAD-MSCs (Bonomi et al., 2013). It seems AD-MSCs are more resistant than BM-MSCs to genotoxic damage of anticancer agents.

Human dental-derived MSCs are resistant to paclitaxel, doxorubicin, and gemcitabine (Brini et al., 2016; Coccè et al., 2017; Salehi et al., 2018). Dental pulp stem cells showed higher resistance to paclitaxel than hBM-MSCs (Salehi et al., 2018). Among the three mentioned drugs, GinPa-MSCs are more resistant to gemcitabine than paclitaxel and doxorubicin. However, all three drugs induce only 20% cytotoxic effect on gingival stem cells at concentrations up to 10,000 ng/mL (Coccè et al., 2017, 2019).

Up to 4,000 ng/mL of paclitaxel did not alter the viability of MSCs from olfactory bulbs (Marei et al., 2019). Placenta-derived hAM-MSCs exhibited high resistance to paclitaxel even at concentrations up to 10,000 ng/mL with a viability more than 90% (Bonomi et al., 2015a). These reports show that the type of MSC and type of anticancer drug are two factors that determine the viability of drug-loaded MSCs. Regardless of concentrations used to evaluate the effect of chemotherapeutic drugs on viability of MSCs, attention to potency and IC_50_ of each chemotherapeutic drug is necessary to load and compare their effects on MSCs, which merits evaluation in the future studies.

#### Mechanisms of Chemotherapeutic Drugs Resistance of MSCs

A variety of probable mechanisms have been reported for MSCs chemotherapeutic drug resistance. The first mechanism involved in chemotherapeutic drug resistance of MSCs is through augmentation of a specific group of agents called heat shock proteins (HSPs) and their genes. Heat shock proteins appear when cells are exposed to physiological and environmental stress to protect the cell against apoptosis. Heat shock proteins also participate in protein folding, transportation of protein, cell cycle regulation, and intracellular signaling (Jego et al., 2013). Cisplatin–pre-exposed hBM-MSCs showed an increased amount of mRNAs such as HSP90AA1, HSP90AB1 (encoding HSP-90 α and β), HSPA1A (encoding HSP-72), HSPB1 (encoding HSP-27), HSPD1 (encoding HSP-60), and HSPE1 (encoding HSP-10), which pose HSPs as one of the MSCs resistance mechanisms (Nicolay et al., 2016a).

Tubulin proteins are the second mechanism for drug resistance in MSCs. Microtubules are critical structures in cell division, movement, and intracellular trafficking. These structures consist of αβ heterodimers, which are the target of some chemotherapeutic drugs (Borisy et al., 2016). Isotypes of tubulin confer chemotherapeutic drug resistance to MSCs by the difference in their drug-binding capacity and dynamicity. In hBM-MSCs, taxol treatment resulted in higher expression of acetylated tubulins, β-III and β-IV (Polioudaki et al., 2009). Taxol binding to β-III and β-IV is weaker than other isotypes of tubulin (Derry et al., 1997); thus, the higher expression of these two isotypes relative to the other tubulins can be one of the resistance mechanisms to taxol in MSCs. Moreover, β-III tubulin isotype forms more dynamic microtubules during mitosis of MSCs, and its hyperdynamicity inhibits taxol effects on the division process (Stengel et al., 2010).

The inhibition of apoptosis in MSCs is the next resistance mechanism. Suppression of P73-dependent proapoptotic pathway, TRAIL, and overexpression of antiapoptotic factors Bcl2 and Bcl-xL are mechanisms involved in the inhibition of apoptosis (Münz et al., 2018). MSCs of dental pulp treated with paclitaxel showed no translocation of cytochrome C enzyme from mitochondria, which means that these cells did not go through apoptosis after exposure to paclitaxel (Salehi et al., 2018).

As the last mechanism of resistance, paclitaxel treatment resulted in increased expression of macrophage migration inhibitory factor. Migration inhibitory factor as an MSC survival promoter induces doxorubicin resistance in hBM-MSCs through activation of PI3K-Akt survival signaling pathway (Xia and Hou, 2018).

There are two other mechanisms of resistance in non-mesenchymal cells, which are not true about MSCs: drug-inactivating system and ATP-binding cassette transporters (ABC transporters). Lack of these mechanisms shows that MSCs do not deactivate or outpour loaded drugs considerably. This feature makes MSCs capable of delivering chemotherapeutic drugs without reducing their cytotoxic function.

Two enzymes contribute to the metabolism of gemcitabine; deoxycytidine kinase and deoxycytidine deaminase (dCDA) as main activating and inactivating enzymes of gemcitabine, respectively (Pessina et al., 2015). As a prodrug, gemcitabine must be metabolized to its active form in MSCs to inhibit proliferation of cancer cells (Amrutkar and Gladhaug, 2017). Released gemcitabine from drug-loaded hBM-MSCs inhibits the proliferation of squamous cell carcinoma of the tongue and pancreatic carcinoma. Considering this inhibitory effect, not only the drug is not inactivated by dCDA, but it is also activated by deoxycytidine kinase (Bonomi et al., 2015b, 2017b). In hBM-MSCs and Hu-OBNSCs, paclitaxel is metabolized to several metabolites, the most abundant of them is 6-α-hydroxyl paclitaxel; however, these metabolites are so slight, which can be ignored (Pascucci et al., 2014; Marei et al., 2019). This confirms that paclitaxel conserves its cytotoxic effect during uptake and release procedure without being extensively metabolized (Salehi et al., 2018).

ABC is a transporter system superfamily involved in the exchange of a variety of substances such as xenobiotic, antibiotics, and chemotherapeutic drugs across biological membranes. Only prokaryotes benefit from the influx (uptake) function of these proteins, whereas efflux property exists in both prokaryotes and eukaryotes (Nobili et al., 2019). *P*-glycoprotein (P-gp) efflux pump as a member of ABC superfamily can induce chemotherapeutic drug resistance in various cells, but it is not the major method of resistance in normal MSCs (Barbet et al., 2012; Lin et al., 2020; Zhang Y. H. et al., 2020). It is recently suggested that inhibition of efflux pumps by verapamil cannot decrease MSCs’ resistance to paclitaxel. In addition, paclitaxel can even down-regulate the expression of P-gp in MSCs (Pessina et al., 2011; Bosco et al., 2015). Other resistance mechanisms cannot be excluded, and further studies should be done to elucidate them.

#### Effect of Chemotherapeutic Drugs on Proliferation of MSCs

The main reason to investigate the proliferation of MSCs after chemotherapeutic drug loading is that proliferation of primed MSCs results in drug content depletion of each cell and thus insufficient drug concentration in the tumor microenvironment. Moreover, MSCs similar to the other stem cells possess self-renewal ability, which can result in tumorigenesis in the proper microenvironment. Thereby, the antiproliferation effect of chemotherapeutic drugs can reduce tumorigenesis and preserve sufficient drug in each loaded MSC.

So far, almost all studies demonstrated that chemotherapeutic agents significantly reduce the proliferation capacity of MSCs in a dose-dependent manner. Because changes in the proliferation of MSCs has a pivotal role in drug loading, it is necessary to address the effect of chemotherapeutic drugs on cell cycle. It has been shown that treatment with paclitaxel (Pessina et al., 2011; Bonomi et al., 2017b; Petrella et al., 2017; Münz et al., 2018), gemcitabine (Bonomi et al., 2015b), pemetrexed (Petrella et al., 2017), bortezomib (Bonomi et al., 2017b), cytarabine, daunorubicin, and vincristine (Somaiah et al., 2018) inhibits cell proliferation of hBM-MSCs. Chemotherapeutic drugs decrease the proliferation of hAD-MSCs. As an instance of this inhibitory effect, proliferation of these cells was decreased by 46% after exposure to paclitaxel (Harris et al., 2017). Actually, 2,000 ng/mL of paclitaxel induced complete cell cycle arrest with minimal cytotoxic effect in hAD-MSCs (Bonomi et al., 2013; Choron et al., 2015). Paclitaxel also reduces DNA synthesis of hAD-MSCs by 80% (Choron et al., 2015). Treatment of GinPa-MSCs leads to an increase in the number of cells in G2/M phase (Coccè et al., 2019).

Paclitaxel-treated hAD-MSCs retrieved their cell growth ability after 5 days; however, full recovery of proliferation capacity was never achieved (Harris et al., 2017). Depending on drug type, the accumulation rate is different in each phase of cell cycle. The majority of hBM-MSCs exposed to paclitaxel are arrested in S phase (Pessina et al., 2011). Exposure to gemcitabine causes arrest of 74% of hBM-MSCs in G0/G1 phases (Bonomi et al., 2015b), and cisplatin induces prolonged arrest of hBM-MSCs in G2 phase (Nicolay et al., 2016a).

Several studies have been done to identify the mechanism of antiproliferative effect of chemotherapeutic drugs in MSCs. High expression of P53 as a cell cycle regulator was reported in vincristine-, cisplatin-, and etoposide-treated MSCs (Polioudaki et al., 2009). Furthermore, higher expression of P53 was observed depending on the dose of taxol or nocodazole. Taxol or nocodazole 500 nM increases P53 permanently in hBM-MSCs, whereas 10 nM of the drugs increases P53 expression proportionately with treatment time (Polioudaki et al., 2009). Growth arrest–specific 1 (GAS1) is a critical regulator of the cell cycle and induces quiescence by preventing cells from entering into S phase. It has been shown that treatment of hBM-MSCs with paclitaxel leads to an increase in GAS1 expression and induces quiescent state (Bosco et al., 2015). In addition, a higher amount of senescence-associated β-galactosidase after paclitaxel treatment suggests that premature senescence is a critical mechanism of MSCs to avoid proliferation and preserve metabolic viability (Münz et al., 2018).

#### Effect of Chemotherapeutic Drugs on Regenerative Capacity of MSCs

Besides the drug delivery ability of MSCs, they play a positive role in chemotherapy-induced tissue damage as a regenerative factor. MSCs are administered to induce postchemotherapeutic tissue regeneration in many organs including kidney (Zoja et al., 2012), hematopoietic system (Koç et al., 2000), lung (Xu et al., 2015), heart (Pinarli et al., 2013), ovary (Badawy et al., 2017), and testis (Sherif et al., 2018). In addition to the ability of MSCs to produce paracrine signals that support progenitors to regenerate chemotherapy-induced tissue damage, they participate in tissue regeneration through other mechanisms such as prevention of inflammation and apoptosis, inducing antioxidative effect, and differentiation to specific cell types in injured organs (Pinarli et al., 2013; Zimmerlin et al., 2013; Sherif et al., 2018). Whether MSCs preserve their initial regenerative characteristics is another important issue that should be evaluated after loading of MSCs with anticancer drugs. A variety of data suggest that the differentiation ability of drug-loaded MSCs as a regenerative mechanism depends on drug and MSC types.

Because BM-MSCs mainly preserve their skeletal differentiation ability after exposure to antineoplastic drugs, they can be preferential to deliver chemotherapeutic agents to skeleton-derived tumors. For example, bleomycin- and paclitaxel-treated hBM-MSCs preserve chondrogenic and osteogenic differentiation capability, respectively (Nicolay et al., 2016b; Münz et al., 2018). On the other hand, some types of MSCs are susceptible to drug-induced differentiation impairment, but they can recover differentiation ability after a drug washing period. These types of MSCs can cause delayed tissue regeneration during the postchemotherapy period. Human adipose-derived MSCs lose their adipogenic and osteogenic differentiation capacity after treatment with paclitaxel. However, partial recovery of differentiation ability was observed after 3 days of drug removal (Harris et al., 2017).

Some chemotherapeutic agents do not influence the differentiation ability of MSCs; thus, these drugs are better choices to provide reconstructive facilities for injured tissues. For example, cisplatin does not influence adipogenic and osteogenic differentiation potential of hBM-MSCs significantly (Nicolay et al., 2016a). In addition, cisplatin- and camptothecin-treated hAD-MSCs did not display any change in osteogenic and adipogenic differentiation capacity (Liang et al., 2011).

### Chemotherapeutic Drug Uptake Capacity of MSCs

Mesenchymal stem cells can uptake the majority of anticancer drugs from the culture environment (Kalimuthu et al., 2018; Salehi et al., 2018; Li et al., 2019). Considering this ability, simple methods have been used to prime hBM-MSCs with drugs. Incubation of hBM-MSCs with paclitaxel (Pessina et al., 2011, 2013; Petrella et al., 2017), gemcitabine (Bonomi et al., 2015b), and sorafenib (Clavreul et al., 2017) leads to effective drug uptake. In contrast, hBM-MSCs incubated with pemetrexed were unable to internalize sufficient drug for affecting mesothelioma (Petrella et al., 2017). Through a simple exposure method, hAD-MSCs are able to uptake cisplatin, cationic platinum (II)-complex (Rimoldi et al., 2018), and paclitaxel (Bonomi et al., 2013; Pacioni et al., 2017). Human dental-derived MSCs possess the ability to uptake paclitaxel, doxorubicin, and gemcitabine (Brini et al., 2016; Coccè et al., 2017; Salehi et al., 2018). It seems the amount of loaded drug per cell depends on the type of MSCs. Each hBM-MSC can uptake approximately 2.7 pg of paclitaxel per cell, which is equivalent to 8% of total drug in the culture medium of paclitaxel (Pessina et al., 2011), whereas Hu-OBNSCs were able to internalize 0.19 pg/cell of paclitaxel.

Based on drug type, there are three mechanisms to uptake anticancer drugs into MSCs including transporters, simple diffusion, and endocytosis. Gemcitabine as hydrophilic nucleoside analog enters cells by nucleoside transporters. Human concentrative nucleoside transporter 1 (hCNT1) and human equilibrative nucleoside transporter 1 (hENT1) are the main transporters of gemcitabine (Hung et al., 2015). The high expression level of hCNT1 and hENT1 in MSCs, which resulted in higher antiproliferative effect of loaded gemcitabine, suggesting that uptake capacity might be attributed to the expression of some transporters (Bonomi et al., 2015b; Coccè et al., 2017). As the next drug internalization mechanism, simple diffusion could be considered according to the lipophilic nature of paclitaxel (Bosco et al., 2015), docetaxel (Wu et al., 2015), camptothecin (Gupta et al., 2000), and etoposide (Patlolla and Vobalaboina, 2008). Endocytosis processes such as pinocytosis, phagocytosis, and receptor-mediated endocytosis are the next mechanisms of drug uptake. The existence of pinocytotic structures in the cytoplasm of GinPa-MSCs implies that the paclitaxel may be internalized by GinPa-MSCs through pinocytosis. CD14 is mainly expressed in the cells, which play a critical role in phagocytosis action (Devitt et al., 1998). Observation of its expression suggests a phagocytic function of GinPa-MSCs for incorporating drugs (Brini et al., 2016). The high expression levels of endocytosis mediator clathrin in hAD-MSCs showed that drugs might internalize through receptor-mediated endocytosis (Wang X. et al., 2019).

### Chemotherapeutic Drug Release Capacity of MSCs

It is important to produce a chemotherapeutic drug delivery system that is able to release drugs locally and slowly to provide an efficient concentration in the tumor microenvironment and diminish systemic toxicity of drugs. MSCs can release antineoplastic drugs in a time-dependent manner that makes them a desirable drug delivery system. The efficacy of drug release depends on cell and drug type. For example, drug-loaded hBM-MSCs started releasing 1 pg/cell of paclitaxel after 2 h, which increased to 1.7–2.0 pg/cell at 144 h (Pessina et al., 2011). Approximately 20% of incorporated sorafenib was released during the first 4 h, and 60% of the drug was released in 48 h, which shows a biphasic pattern in hBM-MSCs (Clavreul et al., 2017). Human bone marrow–derived MSCs have more capability than hAD-MSCs to release paclitaxel, but both cells are able to release paclitaxel in a time-dependent manner. It has been demonstrated that during the first 24 h, hAD-MSCs released the majority of contained paclitaxel, and only minor amounts of the drug were released during the next 48 and 144 h (Bonomi et al., 2013). Approximately 52% of internalized paclitaxel is released from Hu-OBNSCs 24 h after priming (Marei et al., 2019). Evaluation of release capacity of paclitaxel loaded hAM-MSCs revealed that 59% of the total internalized drug was released after 48 h; however, drug release was continued for 120 h (Bonomi et al., 2015a). The differences among release capacity may reflect different hydroliposolubility of drugs. For example, GinPa-MSCs release 62.6% of paclitaxel, 91.8% of gemcitabine, and 100% of doxorubicin. Paclitaxel possesses higher lipophilicity, which is released in the lower amount, whereas water solubility resulted in a higher release of gemcitabine and doxorubicin (Wong et al., 2006; Pili et al., 2009; Coccè et al., 2017).

**FIGURE 3:**
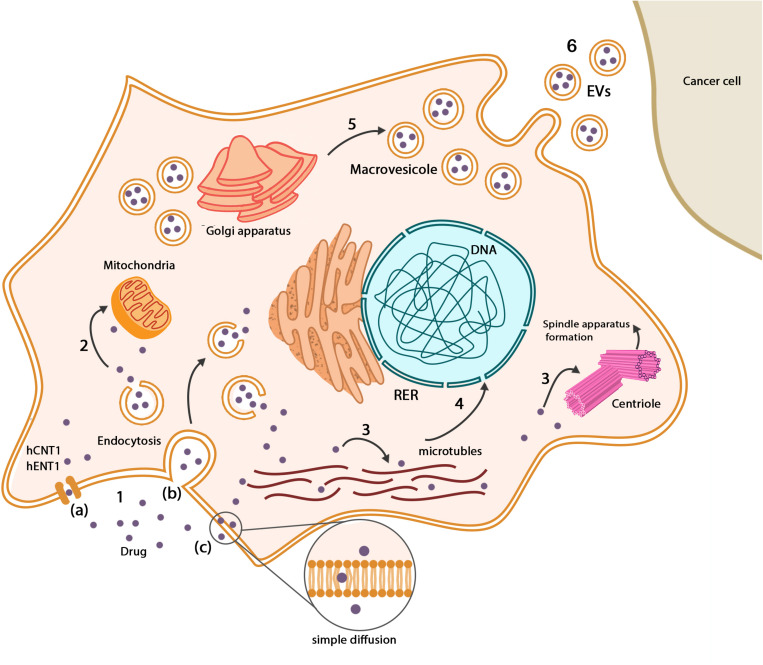
Drugs in MSCs. **(1)** Drugs enter MSCs through a variety of pathways: (a) drug transporters such as hCNT1 and hENT1, (b) endocytosis, (c) simple diffusion based on the chemical nature of chemotherapeutic drugs. **(2)** Some types of drugs such as paclitaxel metabolized in mitochondria, but there is no evidence of impressive inactivation. **(3)** Based on the antineoplastic mechanism of drugs, they are distributed among their place of action such as microtubule networks and centrioles. **(4)** Chemotherapeutic drugs may interfere with the normal gene expression pattern of MSCs, which mainly influence differentiation capacity. **(5)** Mesenchymal stem cells produce vesicles that contain drugs close to the cellular membrane. Drugs can be found in Golgi apparatus and Golgi-derived vesicles. **(6)** Existence of vesicles between MSCs and cancer cells suggests that drugs can be delivered to cancer cells in a vesicular system.

To find the cellular compartments, which are responsible for the storage of drugs, it is necessary to track the anticancer drugs in the MSCs’ membrane and cytoplasm. Paclitaxel can be found along with the microtubule networks, in Golgi apparatus and Golgi-derived vesicles of hBM-MSCs. These vesicles were found close to the cell membrane that explains possible drug release capacity (Pessina et al., 2011; Duchi et al., 2014). Evaluation of GinPa-MSCs showed that multivesicular structures originate from cell membrane budding, and the presence of these structures suggests that GinPa-MSCs may produce exosomes (Brini et al., 2016). At 10,000 ng/mL concentration of cisplatin, hAD-MSCs initiate to form exosomes near cellular membranes (Gilazieva et al., 2016). The secreted vesicles from drug-loaded MSCs contain internalized paclitaxel, which significantly induces antineoplastic effect against ductal pancreatic adenocarcinoma cells. There was no plasma membrane interaction such as gap junction or junctional structure between tumor cells and MSCs (Pessina et al., 2013; Bonomi et al., 2017b), but multiple electron-dense vesicles were observed amid paclitaxel-loaded MSCs and tumor cells (Bonomi et al., 2017b). Studies suggest that MSCs and cancer cells communicate through extracellular vehicles (EVs) which can play pivotal roles both as biological vehicles for drugs and/or endogenous particles. It seems that the transportation system recruits EVs for transferring chemotherapeutic drugs between MSCs and tumor cells (Nawaz et al., 2016; Nawaz, 2017; Fatima and Nawaz, 2017). New methods for transferring of chemotherapeutic drugs by loaded MSCs exist, which herald improvement in drug delivery systems such as ultrasound depletion of drugs (Paris et al., 2017), pH-sensitive nanoparticles, which are released in the tumor microenvironment, visible light–dependent drug release (Gisbert-Garzarán et al., 2016; Martínez-Carmona et al., 2017), and thermal energy as a result of applying magnetic field to release drugs (Guisasola et al., 2015). The uptake and release mechanisms of anticancer drugs in MSCs are shown in Figure 3.

### Antitumor Effect of Drug-Loaded MSCs

Drug-loaded MSCs are applied in two different ways: condition media (CM) and drug carrier cells. Condition media of drug-loaded MSCs contains secretome, which is defined as set of factors secreted to extracellular space. These factors mainly consist of lipids, proteins, free nucleic acids, and EVs. Condition media of drug-loaded MSCs produces more targeted anticancer effect than pure chemotherapeutic drugs. It seems that drug-releasing system of MSCs improve efficacy of loaded drugs through recruiting EVs (Kalimuthu et al., 2018). Application of MSC-sourced CM provides advantages including dosage and potency evaluation, providing storable sources, reduction in invasive cell biopsy procedures, and related safety concerns (Vizoso et al., 2017). Injection of drug-loaded MSCs, which leads to direct cell–cell communication, is another way that causes direct drug transportation between MSCs and cancer cells. Application of the MSCs as a drug carrier is simple and provides a biological sustain release system to deliver chemotherapeutic drugs over a period. Drug-loaded MSCs induce antineoplastic effects through inhibition of proliferation, inducing cytotoxicity against tumor cells, inhibition of angiogenesis and metastasis, and alteration in cytokines secretion of MSCs.

Condition media of drug-loaded hBM-MSCs produces a strong anticancer effect on different cancer cell lines. Condition media of gemcitabine-loaded hBM-MSCs reduced the proliferation of ductal pancreatic adenocarcinoma cells in a concentration-dependent manner (Bonomi et al., 2015b). Condition media of paclitaxel-loaded hBM-MSCs at 1:2 ratio of medium to CM dilution produced 100% growth inhibition in human prostate cancer and glioblastoma cells. This proportion equals 25 ng/mL of pure paclitaxel (Pessina et al., 2011). Condition media of paclitaxel-loaded hBM-MSCs also inhibits proliferation of acute lymphoblastic leukemia, mouse lymphocytic leukemia, malignant pleural mesothelioma, and multiple myeloma cells (Pessina et al., 2013; Bonomi et al., 2017b). Condition media of paclitaxel-treated hAD-MSCs induces a strong dose-dependent antitumor effect on human Ewing sarcoma, human prostate cancer, human blastoma, human neuroblastoma, and acute lymphoblastic leukemia cells. It seems that human Ewing sarcoma cells are more sensitive to paclitaxel in comparison to the other cancer cells (Bonomi et al., 2013). Both CM and lysate of paclitaxel-treated Hu-OBNSCs possess the ability to inhibit glioblastoma and ductal pancreatic adenocarcinoma cells (Marei et al., 2019). Condition media of paclitaxel treated hAM-MSCs can induce dose-dependent antiproliferation effect on ductal pancreatic adenocarcinoma cells (Bonomi et al., 2015a). However, neither CM of pemetrexed- and bortezomib-loaded MSCs, nor lysate of the loaded MSCs, produces antiproliferation activity against malignant pleural mesothelioma, ductal pancreatic adenocarcinoma, or multiple myeloma cells. As mentioned above, hBM-MSCs did not uptake and release pemetrexed and bortezomib sufficiently to produce an antitumor effect (Bonomi et al., 2017b; Petrella et al., 2017). Condition media of GinPa-MSCs exposed to gemcitabine, paclitaxel, and doxorubicin inhibits proliferation of ductal pancreatic adenocarcinoma and squamous cell carcinoma of the tongue (Brini et al., 2016; Coccè et al., 2017).

The coculture of cancer cells with drug-loaded MSCs is used to evaluate the direct antitumor effect of drug-loaded MSCs. Paclitaxel-loaded hBM-MSCs possess the ability to reduce the proliferation of acute lymphoblastic leukemia, glioblastoma, melanoma, and human prostate cancer cells (Pessina et al., 2011, 2013). Coculture of paclitaxel-loaded GinPa-MSCs resulted in significant inhibition of ductal pancreatic adenocarcinoma and squamous cell carcinoma of the tongue cells, whereas non-loaded GinPa-MSCs did not influence cancer cells growth (Coccè et al., 2019). Drug-loaded MSCs are also able to play an anticancer role in animal models. Cotransplantation of acute lymphoblastic leukemia and paclitaxel-loaded hBM-MSCs completely block the formation of tumors in immunodeficient nude mice. In addition, the administration of pure paclitaxel or non-loaded MSCs did not entirely block subcutaneous tumorigenesis of acute lymphoblastic leukemia, whereas intratumoral injection of paclitaxel-loaded MSCs considerably reduced tumor size and weight. Intraperitoneal injection of paclitaxel-loaded MSCs improved survival of mice with lymphocytic leukemia whereas pure paclitaxel administration did not influence the prognosis (Pessina et al., 2013).

Drug-loaded MSCs can reduce angiogenesis in different ways. The proliferation of human umbilical vein endothelial cells was inhibited by CM of sorafenib-treated MSCs (Clavreul et al., 2017). The CM of paclitaxel-loaded hBM-MSCs inhibits VEGF-α which is the major mediator of tumor angiogenesis (Pessina et al., 2011). Intratumoral injection of paclitaxel loaded MSCs to nude mice with subcutaneous acute lymphoblastic leukemia resulted in a reduction of tumor vascularization, microvascular density, and expression of angiogenic markers such as von Willebrand factor, CD31 (PECAM1), VEGF-α, Ve-cad, TGF-β1, CD44, and αSMA. The tumor vascular density of mice treated by paclitaxel-loaded MSCs was reported four times lower than non-loaded MSCs, which indicates higher efficacy of loaded MSCs (Pessina et al., 2013).

Metastasis of cancer cells is a critical problem in cancer treatment and is associated with recurrence and poor prognosis. It has been discovered that CM of paclitaxel loaded hBM-MSCs considerably down-regulates ICAM1 and VCAM1 on TNF-α–activated human microvascular endothelial cells. This down-regulation diminishes the ability of leukemic cells to spread through bloodstream and reduces metastasis chance (Pessina et al., 2013).

Recently, novel administration approaches have been developed; for example, intranasal administration of sorafenib-loaded hBM-MSCs impedes angiogenesis and reduces the number of large vessels (Clavreul et al., 2017). The antineoplastic effect of drug-loaded MSCs with different anticancer drugs and the mechanism of actions in cancer cells are categorized in Table 1.

**TABLE 1:** Anticancer mechanisms of drug-loaded MSCs with a variety of chemotherapeutic drugs.

**Drug**	**Source of MSCs**	**Tumor cells**	**Antitumor effect (ref)**
Doxorubicin	GinPa-MSCs	CFPAC-1 SCC154	Cell cycle arrest (Coccè et al., 2017)
Gemcitabine	hBM-MSCs	CFPAC-1	Cell cycle arrest (Pessina et al., 2015)
	GinPa-MSCs	CFPAC-1 SCC154	Cell cycle arrest (Coccè et al., 2017)
	Pancreas-derived MSCs	CFPAC-1	Cell cycle arrest (Pessina et al., 2015)
Paclitaxel	hBM-MSCs	DU145 T98G MOLT-4 L1210 MPM RPMI8226 B16	Cell cycle arrest, cytokine mediate, cytotoxicity, and antiangiogenesis (Pessina et al., 2011, 2013, 2015; Bonomi et al., 2017b)
	hAD-MSCs	SK-ES-1 DU145 GI-LI-N SH-SY5Y (+) MOLT-4 U87MG	Cell cycle arrest and cytotoxicity (Bonomi et al., 2013; Pacioni et al., 2017)
	GinPa-MSCs	CFPAC-1 SCC154	Cell cycle arrest (Coccè et al., 2017, 2019)
	DPSCs	MCF-7	Cell cycle arrest (Salehi et al., 2018)
	Hu-OBNSCs	U87GM CFPAC-1	Cell cycle arrest (Marei et al., 2019)
	AM-MSCs	CFPAC-1	Cell cycle arrest (Bonomi et al., 2015a)
	SR4987(murine bone marrow-derived MSCs)	MOLT-4 U87MG CFPAC-1 J3T T98G	Cell cycle arrest and antiangiogenesis (Pessina et al., 2011; Pascucci et al., 2014; Pacioni et al., 2015; Bonomi et al., 2017a)
Sorafenib	hBM-MSCs	U87MG	Cytotoxic *in vitro* and antiangiogenesis *in vivo* (Clavreul et al., 2017)

*CFPAC-1, ductal pancreatic adenocarcinoma; SCC154, squamous cell carcinoma of the tongue; MPM, malignant pleural mesothelioma; DU145, human prostate cancer; T98G, glioblastoma cell line; MOLT-4, acute lymphoblastic leukemia; L1210, mouse lymphocytic leukemia; B16, murine melanoma; RPMI8226, multiple myeloma; SK-ES-1, human Ewing sarcoma; SH-SY5Y (+), human neuroblastoma; GI-LI-N, human blastoma; MCF-7, breast cancer; J3T, canine glioma; U87MG, glioblastoma.*

## Conclusion

In recent years, rapid progression in the application of MSCs as a novel treatment for cancer has attracted much interest. MSCs are able to home into the tumor and metastatic sites and change the behavior of cancer and immune cells. They can induce apoptosis and inhibit the proliferation of tumor cells, which are critical points in cancer treatment. In addition, MSCs alter the secretion pattern of cells existing in the tumor microenvironment that can reduce angiogenesis and metastasis. Considering the high resistance of MSCs to a vast majority of antineoplastic agents, MSCs can play a role as a vehicle for targeted drug delivery. Besides this critical characteristic, MSCs can easily uptake chemotherapeutic drugs and release them in a time- and concentration-dependent manner. Both CM and direct contact of primed MSCs induce a considerable antineoplastic effect in a variety of cancer cell lines. Moreover, *in vivo* studies suggest that these drug-loaded cells can reduce the size of tumors and inhibit angiogenesis. Considering promising antineoplastic features of MSCs and drug-loaded ones, MSCs can be a proper candidate to be recruited in the clinic.

## Author Contributions

AB, PS, EJ, and HN contributed to the conception. AB, PS, and EJ reviewed the manuscript and wrote the original draft. HN and MF contributed to the manuscript revision and editing. All the authors read and approved the submitted version.

## Conflict of Interest

The authors declare that the research was conducted in the absence of any commercial or financial relationships that could be construed as a potential conflict of interest.

## Abbreviations

AM-MSCs: amniotic membrane-derived mesenchymal stem cells
dCDA: deoxycytidine deaminase
GinPa-MSCs: gingival-derived mesenchymal stem cells
hAD-MSCs: human adipose-derived mesenchymal stem cells
hBM-MSCs: human bone marrow-derived mesenchymal stem cells
hD-MSCs: human dental-derived mesenchymal stem cells
Hu- OBNSCs: human olfactory bulb neural stem cells
MMP: matrix metalloproteinases
MMP: mitochondrial membrane potential
MSCs: mesenchymal stem cells.
